# Long-term follow-up including extensive complement analysis of a pediatric C3 glomerulopathy cohort

**DOI:** 10.1007/s00467-021-05221-6

**Published:** 2021-09-02

**Authors:** Marloes A. H. M. Michels, Kioa L. Wijnsma, Roel A. J. Kurvers, Dineke Westra, Michiel F. Schreuder, Joanna A. E. van Wijk, Antonia H. M. Bouts, Valentina Gracchi, Flore A. P. T. Engels, Mandy G. Keijzer-Veen, Eiske M. Dorresteijn, Elena B. Volokhina, Lambertus P. W. J. van den Heuvel, Nicole C. A. J. van de Kar

**Affiliations:** 1 Department of Pediatric Nephrology, Amalia Children’s Hospital, Radboud University Medical Center, Radboud Institute for Molecular Life Sciences, Nijmegen, The Netherlands; 2 Department of Pediatric Nephrology, Maastricht University Medical Center, Maastricht, The Netherlands; 3 Department of Pediatric Nephrology, Emma Children’s Hospital, Amsterdam University Medical Centers, Amsterdam, The Netherlands; 4 Department of Pediatric Nephrology, Beatrix Children’s Hospital, University Medical Center Groningen, University of Groningen, Groningen, The Netherlands; 5 Department of Pediatric Nephrology, Wilhelmina Children’s Hospital, University Medical Center Utrecht, Utrecht, The Netherlands; 6 Department of Pediatric Nephrology, Sophia Children’s Hospital, Erasmus Medical Center, Rotterdam, The Netherlands; 7 Department of Laboratory Medicine, Radboud University Medical Center, Nijmegen, The Netherlands; 8 Department of Pediatrics/Pediatric Nephrology and Department of Development and Regeneration, University Hospitals Leuven, Leuven, Belgium

**Keywords:** Children, C3 glomerulopathy, Complement system, C3 nephritic factor, Dense deposit disease, C3 glomerulonephritis

## Abstract

**Background:**

C3 glomerulopathy (C3G) is a rare kidney disorder characterized by predominant glomerular depositions of complement C3. C3G can be subdivided into dense deposit disease (DDD) and C3 glomerulonephritis (C3GN). This study describes the long-term follow-up with extensive complement analysis of 29 Dutch children with C3G.

**Methods:**

Twenty-nine C3G patients (19 DDD, 10 C3GN) diagnosed between 1992 and 2014 were included. Clinical and laboratory findings were collected at presentation and during follow-up. Specialized assays were used to detect rare variants in complement genes and measure complement-directed autoantibodies and biomarkers in blood.

**Results:**

DDD patients presented with lower estimated glomerular filtration rate (eGFR). C3 nephritic factors (C3NeFs) were detected in 20 patients and remained detectable over time despite immunosuppressive treatment. At presentation, low serum C3 levels were detected in 84% of all patients. During follow-up, in about 50% of patients, all of them C3NeF-positive, C3 levels remained low. Linear mixed model analysis showed that C3GN patients had higher soluble C5b-9 (sC5b-9) and lower properdin levels compared to DDD patients. With a median follow-up of 52 months, an overall benign outcome was observed with only six patients with eGFR below 90 ml/min/1.73 m^2^ at last follow-up.

**Conclusions:**

We extensively described clinical and laboratory findings including complement features of an exclusively pediatric C3G cohort. Outcome was relatively benign, persistent low C3 correlated with C3NeF presence, and C3GN was associated with higher sC5b-9 and lower properdin levels. Prospective studies are needed to further elucidate the pathogenic mechanisms underlying C3G and guide personalized medicine with complement therapeutics.

**Graphical abstract:**

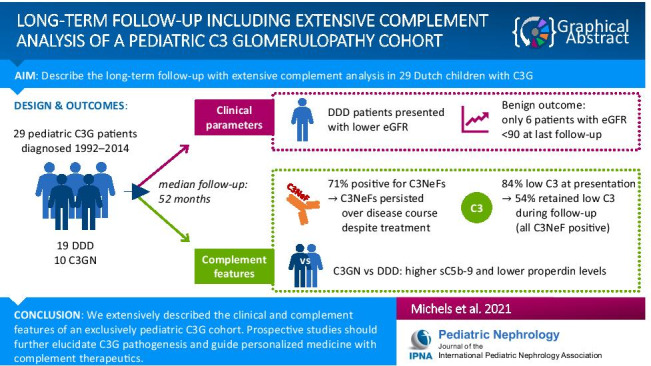

**Supplementary Information:**

The online version contains supplementary material available at 10.1007/s00467-021-05221-6.

## Introduction

Over the last decade, the role of the complement system in many kidney disorders has been elucidated. C3 glomerulopathy (C3G) is one of these disorders that could be further characterized by increased knowledge of the underlying (complement) pathophysiology. C3G is a rare and severe kidney disease caused by dysregulation of the complement alternative pathway (AP) [1–3]. Although several clinical symptoms, such as proteinuria, hematuria, and hypertension, possibly combined with low serum C3 levels, can be indicative of C3G, the diagnosis of C3G is still solely based on kidney biopsy. The defining characteristic in the kidney biopsy is the dominant immunofluorescence staining for C3 depositions in the glomerulus of at least two orders of magnitude greater than the intensity for other immune reactants [1]. Based on electron microscopy findings, C3G is further subdivided into its two major entities: dense deposit disease (DDD) and C3 glomerulonephritis (C3GN) [1, 3].

In addition to the prominent glomerular C3 staining in kidney biopsies, the presence of autoantibodies known as C3 nephritic factors (C3NeFs) is indicative of the AP dysregulation in C3G. In 50–80% of C3G patients, these C3NeFs can be found [4–7]. C3NeFs prolong the activity of the AP convertases, leading to an overactive complement system via increased C3 conversion [8]. Low serum C3 levels indicate complement consumption in the majority of patients, but also other complement components and complement activation products may show an abnormal yet highly heterogenous pattern in patients with C3G [9]. In addition, in up to 20% of C3G cases, genetic aberrations are found in AP complement (regulatory) genes [4, 7, 10].

Since C3G is associated with a poor prognosis and high rate of recurrence after kidney transplantation, targeted therapy is needed. In recent years, a sharp increase in the development of complement-directed therapeutics can be noted, but many still have to pass the clinical trials [11]. Several phase II clinical trials with different complement inhibitors are currently including C3G patients [12]. Eculizumab, a C5 inhibitor and the first complement therapeutic drug that gained market approval, has proven its value in the treatment of another complement-mediated kidney disease, i.e., atypical hemolytic uremic syndrome. Although some case studies report a positive clinical response, the benefit of eculizumab treatment in patients with C3G remains unproven [13–15].

In light of these (future) therapeutic opportunities, it has become very important to fully characterize the patient with C3G [3]. Recent advances in the development of assays measuring markers of complement activation and C3NeF types enable such improved patient characterization. Moreover, a recent study has shown that the incorporation of complement biomarker analyses, in addition to clinical and pathological characteristics, can be used to identify novel subgroups of C3G with distinct pathogenic patterns [16]. Furthermore, complement biomarkers such as C3 are increasingly used as inclusion criteria and/or as outcome measures of clinical trials with novel complement-targeted therapies.

Most of the cohort studies of patients with C3G comprise a mixed and thus heterogeneous group of both children (mostly adolescents) and adults [4, 7, 10, 17–20]. Only a few studies on C3G in a pediatric cohort have been published [21–26]. In this study, we describe the long-term follow-up of 29 Dutch children with C3G, including clinicopathological features, complement aberrations, and complement biomarker profiles.

## Methods

### Patient inclusion

In this retrospective observational longitudinal cohort study, all children (0–18 years of age) were included who were diagnosed with C3G in one of five university medical centers in the Netherlands between December 1, 1992, and April 14, 2014, and from whom samples were sent in for complement diagnostics to the unit Complement Mediated Disorders of the Translational Metabolic Laboratory of the Radboudumc.

Date of presentation was defined as the first intake or admission at one of five medical centers. Diagnosis was suspected on clinical features such as hypertension and/or proteinuria and/or nephrotic/nephritic syndrome, possibly combined with low serum C3 levels, and was confirmed by pathological findings at kidney biopsy according to the consensus statement defined in the C3 Glomerulopathy Meeting [1]. Five patients were treated with eculizumab during follow-up and were described separately by Oosterveld et al. [27].

The study has been reviewed and was approved by the ethics committee on the basis of the Dutch Code of Conduct for Medical Research, the Dutch Personal Data Protection Act, and the Medical Treatment Agreement Act (2014–1334).

### Collection of clinical data and standard laboratory measurements

Demographic, clinical, and standard laboratory data at the time of diagnosis were obtained from the patients’ medical records. Follow-up data, when available, were collected at 6 and 12 months and at 2, 4, 6, 8, and 10 years after diagnosis until patients reached the age of 18 years. Hypertension was defined as a systolic blood pressure and/or diastolic blood pressure equal to or greater than the 95th percentile corrected for age, gender, and height. Proteinuria was defined as a protein/creatinine ratio > 22 mg/mmol. Nephrotic syndrome was diagnosed in the presence of heavy proteinuria (protein/creatinine ratio ≥ 200 mg/mmol), hypoalbuminemia (≤ 25 g/L), and clinical edema. Hematuria was diagnosed when there were ≥ 5 erythrocytes/high-powered field. The estimated GFR (eGFR) was calculated with the Schwartz formula (k: 36.5, serum creatinine level expressed as µmol/l) [28]. An impaired eGFR was defined as an eGFR < 90 ml/min/1.73 m^2^. Kidney injury was defined as hypertension and/or proteinuria and/or eGFR below 90 ml/min/1.73 m^2^. The number of relapses was documented during follow-up and defined as clinical and biochemical deterioration with the need to intensify the medical regimen.

### Genetic analysis

Genomic DNA, isolated from peripheral blood leukocytes, was amplified for *CFH* (NM_000186.3), *CFHR1* (NM_002113.2), *CFHR2* (NM_005666.2), *CFHR3* (NM_021023.5), *CFHR4* (NM_001201550.2), *CFHR5* (NM_030787.3), *CFI* (NM_000204.3), *CD46* (NM_002389.4), *C3* (NM_000064.2), and *CFB* (NM_001710.5) by means of PCR, followed by sequence analysis and screening for rare genetic variants (minor allele frequency < 1%) [29]. In addition, multiplex ligation-dependent probe amplification (MLPA) was used to screen for rearrangements in the *CFH/CFHR* region.

### Specialized complement analyses

Enzyme-linked immunosorbent assay (ELISA) techniques were used to determine the levels of the complement activation markers C3bBbP (properdin-stabilized AP C3 convertase), C3bc (sum of C3 activation products C3b, iC3b, and C3c; together with C3bBbP marker for proximal complement cascade activation), and soluble C5b-9 (sC5b-9; end product of complement activation), the positive AP regulator properdin, the negative complement regulators Factor H (FH) and Factor I (FI), and the complement component C5 in serum or ethylenediamine-tetra-acetic acid (EDTA)-plasma samples, as described previously [30–33]. These assays were performed on patient samples available in the − 80 °C storage. If available, samples from multiple collection dates within the study period, i.e., from presentation up to last follow-up, were analyzed.

Samples were tested for the presence of C3NeF using a hemolytic convertase activity assay described previously [34]. Patients were considered as positive if one of their samples tested positive at least twice for prolonged convertase activity. Samples were tested for the presence of autoantibodies against FH by means of an ELISA based on Dragon-Durey et al. [35].

### Statistical methods

Statistical analysis was performed using SPSS (version 25, SPSS, Inc.). Values are expressed using median and interquartile range (IQR) for continuous variables and percentages for qualitative variables. To analyze differences between continuous variables, the Mann–Whitney *U* test was used. Qualitative variables were compared using the chi–square or Fisher’s exact test.

Using R and R studio with the package lme4 [36], a linear mixed effects model was performed on the different complement markers C3bBbP, C3bc, sC5b-9, C5, properdin, FH, and FI, taking all measurements across time into account. Diagnosis was used as a fixed factor, and random intercepts were used for each subject to account for the potential correlation within subjects with multiple measurements.

Differences were considered statistically significant at a *P*-value of less than 0.05.

## Results

### Patient characteristics at presentation

During the study, 29 children were diagnosed with C3G in five university medical centers in the Netherlands. Kidney biopsies, available of all patients, by definition showed dominant C3 staining by immunofluorescence microscopy in all cases. Of these 29 patients, 19 (65%) were classified as DDD and ten (35%) as C3GN. A small majority was female (62%). The median (IQR) age at presentation was 7 (6.0–8.5) years, with DDD patients presenting at a slightly younger age than C3GN patients (*P* = 0.046; Table 1). The majority of the patients presented with proteinuria (93%) and/or hematuria (90%), and more than half of the patients had hypertension (64%). In 12 cases (41%), an infectious trigger was identified, of which six were upper respiratory infections, five lower respiratory infections (two cases of mycoplasma pneumonia), and one urinary tract infection. In addition, one DDD patient presented with signs of partial lipodystrophy. In total, 18 (62%) patients presented with an impaired eGFR and 17 (59%) with decreased serum albumin levels. Fourteen (48%) patients fulfilled the criteria of a nephrotic syndrome. The median eGFR (IQR) at presentation was 74 (49–112) ml/min/1.73 m^2^ with a significantly lower eGFR in the patients with DDD versus C3GN (*P* = 0.022; Table 1).

**Table. 1:** Patient characteristics at presentation

	Total (*n* = 29)	DDD (*n* = 19)	C3GN (*n* = 10)	*P*-value
Clinical features				
**Age (year)**	7 (6.0–8.5)	6 (6.0–7.0)	8 (6.8–10.0)	0.046
**Female**	18 (62%)	12 (63%)	6 (60%)	NS
**Infectious trigger**	12 (41%)	7 (37%)	5 (50%)	NS
**Hypertension**	16/25 (64%)	10/16 (63%)	6/9 (67%)	NS
**eGFR (ml/min/1.73 m**^**2**^**)**	74 (49–112)	69 (20–85)	103 (73–132)	0.022
*eGFR* < *90 ml/min/1.73 m*^*2*^	*18 (62%)*	*15 (79%)*	*3 (30%)*	*0.017*
**Hematuria**	26 (90%)	18 (95%)	8 (80%)	NS
**Nephrotic syndrome**^a^	14 (48%)	10 (53%)	4 (40%)	NS
*Proteinuria (mg/mmol)*	*481 (195–965);n* = *24*	*492 (266–1008);n* = *15*	*470 (42–957);n* = *9*	*NS*
*Serum albumin(reference range:34–55 g/l)*	*28 (21–40)*	*25 (21–38)*	*34 (20–41)*	*NS*
Complement investigation*Reference range*				
**C3 levels*** 700–1500 mg/l*	220 (95–548);*n* = 25	400 (150–644);*n* = 15	165 (78–290);*n* = 10	NS
**C3d levels** < *2%*	6.2 (4.4–8.3);*n* = 10	6.1 (5.3–6.9);*n* = 7	7 (0.0–7.0);*n* = 3	NS
**C4 levels*** 100–400 mg/l*	208 (134–283);*n* = 24	260 (171–294);*n* = 15	150 (120–207);*n* = 9	0.014
**C3NeF**	20/28 (71%)	12/18 (67%)	8/10 (80%)	NS
**Factor H autoantibodies**	2/28 (7%)	1/18 (6%)	1/10 (10%)	NS
** Rare genetic variants**	3/27 (11%)	2/17 (12%)	1/10 (10%)	NS
Treatment initiated at presentation				
**Prednisolone**	22 (76%)	15 (79%)	7 (70%)	NS
*Methylprednisolone pulse therapy*	*15 (52%)*	*11 (58%)*	*4 (40%)*	*NS*
**Mycophenolate mofetil**	6 (21%)	3 (16%)	3 (30%)	NS
**Cyclophosphamide**	2 (7%)	2 (11%)	0	NS
**Rituximab**	1 (3%)	0	1 (10%)	NS
**Plasma therapy**^b^	7 (24%)	6 (32%)	1 (10%)	NS
**Eculizumab**	3 (10%)	3 (16%)	0	NS
**Kidney replacement therapy**	2 (7%)	2 (11%)	0	NS

*eGFR* estimated glomerular filtration rate, *C3NeF* C3 nephritic factor, *DDD* dense deposit disease, *C3GN* C3 glomerulonephritis. Categorical values are expressed as absolute number with the percentage of total in parentheses. For continuous variables, the median is expressed with interquartile range in parentheses. In case of missing data, the percentage was calculated using only the patients of whom data was available for analysis

^a^Nephrotic syndrome was diagnosed in the presence of protein/creatinine ratio ≥ 200 mg/mmol, hypoalbuminemia (< 25 g/l), and clinical edema

^b^Plasma therapy comprises both plasma exchange (*n* = 5) and plasma infusions (*n* = 2)

At presentation, various treatment regimens were initiated by the physicians. Prednisolone was used most frequently in a total of 22 (76%) patients; 15 received methylprednisolone pulse treatment prior to start of prednisolone. In six patients, mycophenolate mofetil treatment was started at presentation in addition to prednisolone therapy. Seven patients were treated with plasma therapy and one patient received rituximab. Eculizumab was given to three patients at first presentation, in addition to immunosuppressive and/or plasma therapy. Only two patients, diagnosed as DDD, required temporary kidney replacement therapy, which could be stopped within 6 months.

### Patient outcome at last follow-up

The patients had a median (IQR) follow-up duration of 52 (27–91) months (Table 2). During this follow-up, 13 (45%) patients experienced a relapse. In 11 patients, only one relapse was reported and two patients experienced a second relapse. Of note, 92% (12/13) of the patients experiencing relapses were found positive for C3NeF during their disease course, indicating relapses occurred more often C3NeF-positive patients compared to C3NeF-negative patients (*P* = 0.038). Furthermore, two DDD patients developed ocular drusen during follow-up and one C3GN patient developed partial lipodystrophy.

**Table. 2:** Patient outcome at last follow-up

	All (*n* = 29)	DDD (*n* = 19)	C3GN (*n* = 10)	*P*-value
**Duration follow-up (months)**	51 (26–90)	57 (22–101)	46 (30–69)	NS
Biochemical values*Reference range*				
**eGFR (*n* = 28)** > *90 ml/min/1.73 m*^*2*^	110 (93–12);*n* = 28	107 (82–125);*n* = 18	110 (98–131);*n* = 10	NS
**C3 levels (*n* = 17)** *700–1500 mg/l*	500 (233–871);*n* = 17	351 (233–904);*n* = 9	505 (213–829);*n* = 8	NS
Treatment				
**None**	4 (14%)	2 (11%)	2 (20%)	NS
**RAAS inhibition**^a^	23 (80%)	17 (90%)	7 (70%)	NS
**Immunosuppressive medication**	14 (48%)	7 (37%)	7 (70%)	NS
*Prednisolone*	*8 (28%)*	*6 (32%)*	*2 (20)*	*NS*
*Mycophenolate mofetil*	*10 (35%)*	*4 (21%)*	*6 (60%)*	*0.051*
*Calcineurin inhibitors*	*1 (3%)*	*0*	*1 (10%)*	*NS*
**Eculizumab**^b^	4 (14%)	3 (16%)	1 (10%)	NS
Outcome				
**Relapses**	13 (45%)	8 (42%)	5 (50%)	NS
**Persistent kidney injury**^c^	19 (66%)	12 (63%)	7 (70%)	NS
*Impaired eGFR(*< *90 ml/min/1.73 m*^*2*^*)*	*6 (21%)*	*5 (26%)*	*1 (10%)*	*NS*
*Proteinuria*	*14 (48%)*	*8 (42%)*	*6 (60%)*	*NS*
**Kidney replacement therapy**	1 (3%)	1 (5%)	0	NS

*eGFR* estimated glomerular filtration rate, *RAAS* renin–angiotensin–aldosterone system, *DDD* dense deposit disease, *C3GN* C3 glomerulonephritis. Categorical values are expressed as absolute number with the percentage of total in parentheses. For continuous variables, the median is expressed with interquartile range in parentheses. In case of missing data, the percentage was calculated using only the patients of whom data was available for analysis

^a^RAAS inhibition includes angiotensin-converting enzyme inhibitors as well as angiotensin receptor blockers

^b^At last follow-up, four patients received eculizumab, however in total five patients have received eculizumab during the study period (*n* = 4 DDD, *n* = 1 C3GN)

^c^Persistent kidney injury was defined as hypertension and/or proteinuria and/or an impaired GFR below 90 ml/min/1.73 m^2^

At last follow-up, 22 (79%) patients had an eGFR above 90 ml/min/1.73 m^2^. Only six (21%) patients, of whom five patients with DDD and one with C3GN, had an impaired eGFR (Table 2). Only one patient (DDD) required kidney replacement therapy due to kidney failure. The remaining DDD patients had an eGFR at last presentation of respectively 47, 50, 58, and 89 ml/min/1.73 m^2^, and the patient with C3GN had an eGFR of 87 ml/min/1.73 m^2^. Of note, no proteinuria and hypertension (and no renin–angiotensin–aldosterone system inhibition) were reported in the DDD patient with an eGFR of 89 ml/min/1.73 m^2^. The majority of the patients had an impaired eGFR already within 1 year of follow-up. No statistically significant difference in eGFR at last follow-up was observed between the patients with and without a relapse and with or without persistent kidney injury (data not shown). Despite the good eGFR outcome in the majority of the patients, 14 (48%) patients had proteinuria at last follow-up. Of the 15 patients without proteinuria at last follow-up, proteinuria levels normalized and remained normalized until last follow-up within 6 months in four patients (follow-up of 16.4, 51.8, 59.9, and 100.4 months) and within 12 months in two patients (follow-up of 22.0 and 41.0 months).

The majority (86%) of patients used medication at the last follow-up. Overall, 14 (48%) patients received immunosuppressive medication (Table 2). Of the three patients who received eculizumab at first presentation, treatment was discontinued in one patient within the first 6 months. Two other patients started on eculizumab more than a year after initial presentation. Four patients received eculizumab at last follow-up. No significant difference was observed between patients who received eculizumab treatment and patients who did not regarding eGFR, C3 levels, or range of proteinuria at presentation versus last follow-up (data not shown).

### Complement aberrations linked to development of C3G

**Fig. 1:**
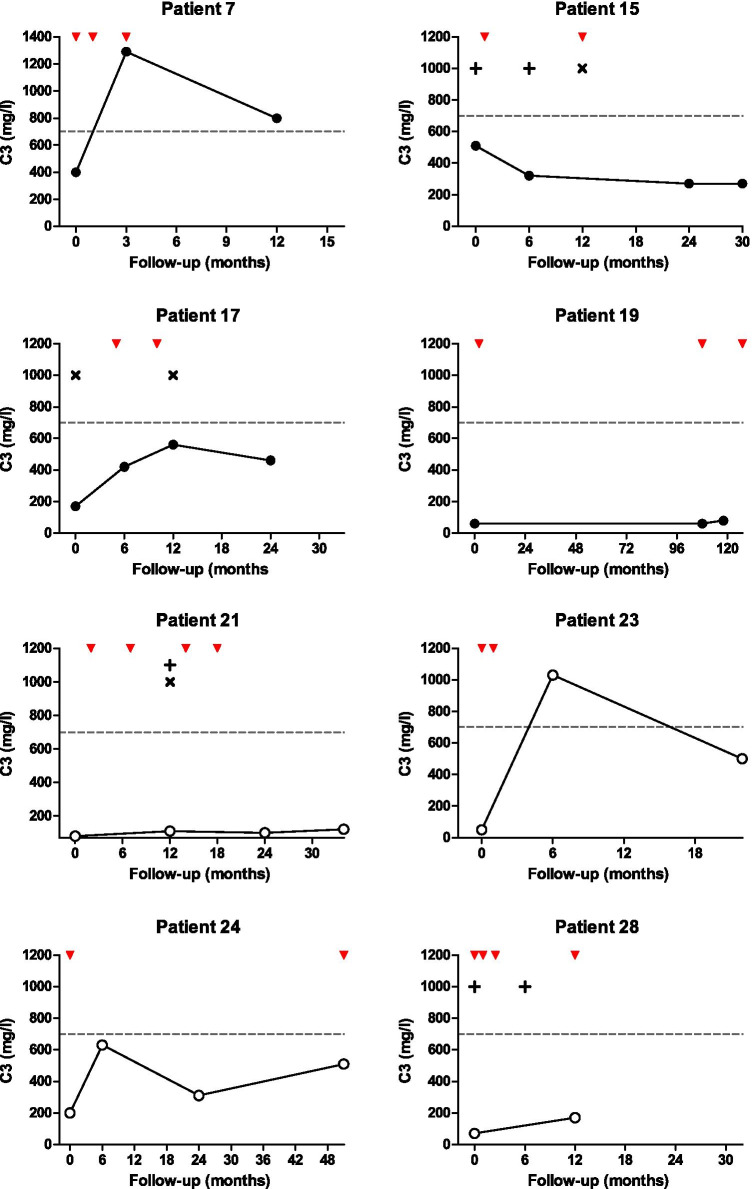
Serial C3 nephritic factor measurements over time in relation to serum C3 levels. In eight patients, C3 nephritic factors (C3NeFs) were detected on multiple occasions during follow-up (indicated by red downward pointing arrowheads) and were correlated to C3 levels in time. Closed dots represent dense deposit disease (DDD, *n* = 4) patients and open dots represent C3 glomerulonephritis (C3GN, *n* = 4) patients. The gray dotted lines indicate the lower cutoff value for normal C3 levels. Length of *x*-axes represents the clinical follow-up time. Plus signs indicate that patients received plasma exchange; multiplication signs indicate that patients received plasma infusion. In two patients (patients 7 and 23), C3 levels normalized during follow-up while patients were treated with prednisolone and mycophenolate mofetil

C3NeFs were found in 20 out of 28 patients (71%) with no difference in the presence of C3NeFs in DDD versus C3GN patients (Table 1). Samples tested for C3NeFs were taken after a median (IQR) of 2.0 (1.0–26) months after presentation, and no significant difference in sampling time was observed between the C3NeF positive and negative group. In addition, for eight patients, serial measurements over time were available (Fig. 1), with a median (range) sampling follow-up time of 10.5 (0.7–125.7) months. During follow-up, C3NeF presence was preserved in all patients despite immunosuppressive medication and/or plasma exchange (Fig. 1). In addition to C3NeFs, autoantibodies against FH were detected in two patients, i.e., patient 15 with DDD and patient 24 with C3GN.

Of the 29 patients included, 27 were screened for rare variants in genes encoding proteins of the AP. In three patients, rare genetic variants were identified in *CFI*, *CFHR2*, and *CFHR5* (Supplementary Table 1). All were classified as variants of uncertain significance or as likely benign (*CFI*). One patient, P13 (DDD), carried a single rare variant, and two patients, P8 (DDD) and P21 (C3GN), carried two rare variants. P13 and P21 additionally carried C3NeFs. There were no familial cases of C3G or *CFHR5* gene variants belonging to CFHR5 nephropathy.

### Serum C3, C3d, and C4 at presentation and during follow-up

At presentation, 21 out of 25 (84%) patients had decreased (< 700 mg/l) C3 levels (Supplementary Fig. 1a) with a median (IQR) of 220 (95–548) mg/l (Table 1). These C3 levels did not differ between the DDD and C3GN group (Table 1), nor did they differ between the groups during or at last follow-up (Table 2). C3d levels were elevated (> 2.0%) in eight out of ten patients at presentation (Supplementary Fig. 1b) with a median (IQR) of 6.2 (4.4–8.3)% (Table 1). C4 levels were above the lower cutoff of 100 mg/l in all patients at presentation (Supplementary Fig. 1c and Table 1), as well as during follow-up (data not shown).

**Fig. 2:**
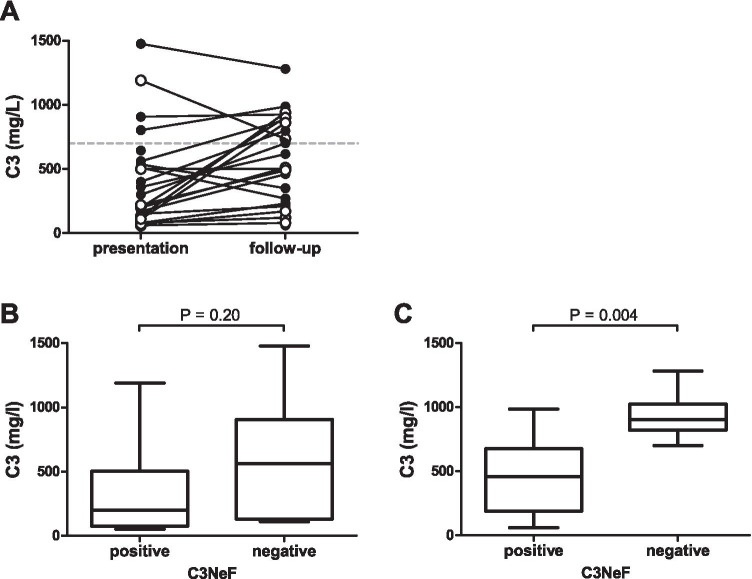
C3 levels at presentation versus last known value at follow-up and in relation to presence of C3 nephritic factors. **A** Closed dots represent dense deposit disease (DDD) patients and open dots represent C3 glomerulonephritis (C3GN) patients. The gray dotted line indicates the lower cutoff value for normal C3 levels. **B**–**C** The distribution of C3 levels measured at presentation (**B**) and the last reported C3 level during follow-up (**C**) according to whether patients were found positive for C3 nephritic factors (C3NeFs) or not. The box plots show the median with the quartiles and the 5th and 95th percentiles (whiskers). *P*-values were calculated with the Mann–Whitney *U* test

Next to the C3 level at last follow-up (Table 2), last reported C3 levels per patient were collected and showed a median (IQR) of 505 (232–877) mg/l. In 13 out of 24 (54%) patients, these C3 levels at last measurement were below the control range (Fig. 2A). Although C3 levels did not differ between DDD and C3GN, we did find differences when patients were categorized based on whether they had tested positive for C3NeFs in the study period or not. The C3 levels at presentation did not differ between the C3NeF-positive and C3NeF-negative group (Fig. 2B; *P* = 0.20), but C3 levels during follow-up were significantly lower in patients who had tested positive for C3NeFs compared to those who tested negative (Fig. 2C; *P* = 0.004). At last measurement, only four out of 17 C3NeF-positive patients had normal C3 levels, whereas the six C3NeF-negative patients all had normal C3 levels.

Moreover, in the eight patients with serial C3NeF measurements, C3 levels were often below control range at the time C3NeFs were detected (Fig. 1). However, in patient 7, C3 levels temporarily normalized despite C3NeF presence and no material of patient 23 was available to test for C3NeFs at time of C3 normalization.

### Complement marker profiles in DDD versus C3GN patients

For 21 patients (*n* = 10 C3GN, *n* = 11 DDD), samples originating from the study period with a median (IQR) collection time after presentation of 13.0 (1.8–46.2) months were available to test for one or more of the following complement biomarkers: C3bBbP, C3bc, sC5b-9, C5, properdin, FH, and FI. To investigate if the complement biomarkers differed between the DDD and C3GN group, all measurements across time and irrespective of the disease state were taken into account in a linear mixed model analysis (Table 3). The samples from patients on eculizumab were excluded from this analysis. We observed significantly higher sC5b-9 levels in the C3GN patients than in the DDD patients (*P* = 0.031). In contrast, properdin levels were significantly lower in patients with C3GN (*P* = 0.043). The other markers were not significantly different between DDD and C3GN patients (Table 3).

**Table. 3:** Mixed model analysis of complement marker profiles in C3GN and DDD patients

Complement marker (reference value)	Measurements (*n*)	Patients (*n*)	C3GN Mean (95% CI)	DDD Mean (95% CI)	Difference between means and 95% CI	*P*-value
C3bBbP (< 12 CAU)	32	17	24.1 (15.6–32.7)	17.3 (9.7–24.9)	6.8 (− 4.4–18.0)	NS
C3bc (< 15 CAU)	32	17	17.0 (9.5–24.5)	18.7 (12.2–25.3)	− 1.7 (− 11.5–8.0)	NS
sC5b-9 (< 0.5 CAU)	33	17	4.0 (1.8–6.2)	0.9 (0^a^–2.7)	3.2 (0.2–6.0)	0.043
C5 (42–93 µg/ml)	44	21	43.5 (28.5–58.5)	58.5 (44.6–72.4)	− 15.0 (35.0–5.0)	NS
Properdin (11.0–30.8 µg/ml)	46	21	17.3 (12.6–21.9)	24.6 (20.3–28.9)	–7.4 (− 13.6 to − 1.2)	0.031
FH (122–315 µg/ml)	43	20	288.2 (236.0–340.3)	288.5 (237.1–339.9)	− 0.3 (− 71.8–71.2)	NS
FI (22–41 µg/ml)	39	18	27.0 (22.0–32.1)	29.2 (24.6–33.8)	− 2.2 (− 9.3–4.6)	NS

*C3GN* C3 glomerulonephritis, *DDD* dense deposit disease, *CI* confidence interval, *CAU* complement arbitrary units, *FH* Factor H, *FI* Factor I, *sC5b-9* soluble C5b-9

^a^For the individual diagnosis types, negative lower limits of the 95% confidence interval (CI) were stated as 0

## Discussion

In this study, we describe the long-term follow-up of a large pediatric C3G cohort, combined with an in-depth complement analysis over time. With our extensive retrospective analyses, we add important information regarding the relation between the presence of C3NeFs and clinical parameters such as C3 levels and disease recurrence. Moreover, C3NeF presence remained detectable over time despite immunosuppressive treatment. Profound analyses showed higher sC5b-9 and lower properdin levels over time in patients with C3GN than in DDD, suggestive of terminal complement pathway activation. Furthermore, in contrast to other cohort studies, a benign outcome was observed and only a few patients had an impaired kidney function.

Various complement aberrations have been described as cause of the complement dysregulation in patients with C3G, with one of the most important being C3NeFs. With our validated functional assay, we detected C3NeFs in 71% of the patients tested, which is consistent with reports in the literature [4–7]. When focusing on the pediatric population only, our findings are in line with those of Marinozzi et al., who reported C3NeFs in 72% of the 57 pediatric patients they describe [37]. In most other cohorts describing the presence of C3NeFs in children, the number of patients tested is relatively low (≤ 11) and the percentage of C3NeF-positive patients ranges between 25 and 100% [13, 21–23, 25, 38]. Some studies have reported C3NeFs more often in DDD than in C3GN [4, 7], but in our pediatric cohort no significant difference was found.

Rare variants in AP complement regulatory genes were found in only three patients, but no (likely) pathogenic genetic variants were identified in our cohort. In two patients with genetic variants, C3NeFs were also detected, leading to the question whether the genetic variation or rather the presence of C3NeF is driving the complement dysregulation in these patients. The role of rare genetic complement variants in C3G is further questioned by a recent study of Levine et al. that did not find enrichments of rare variants in complement (regulatory) genes in C3G patients. Instead, an HLA type was found to be associated with C3G, implicating that the disease should be considered an autoimmune process rather than a primary genetic disorder [39].

Importantly, a positive C3NeF finding in patients was linked to significantly lower C3 levels during follow-up and with disease relapses. Furthermore, C3NeFs remained detectable during follow-up despite immunosuppressive treatment, and their presence was consistently associated with lowered C3 levels in six out of the eight patients. Such longitudinal data on C3NeF presence in association with C3 levels are scarce in the literature [40]. Nonetheless, we as well as others have also shown that a normal C3 level does not exclude the presence of C3NeFs, and that C3NeFs can disappear (spontaneously) during the disease course [4, 26, 40]. Interestingly, we observed that patients who experienced a relapse were significantly more often C3NeF positive. This suggests a continuous pathogenic role of these autoantibodies during the course of disease and this could potentially influence treatment decisions. To further address the prognostic value of C3NeF, prospective data collection, of both clinical and chemical features, will be required. Moreover, one could question the (sub)classification of C3G solely based on pathology and maybe could rather identify novel subgroups of C3G based on the complement biomarker and autoantibody profile [16].

In C3GN patients, a significantly higher sC5b-9 level and lower properdin level was found using a linear mixed model to analyze the complement profiles with the longitudinal data of C3bBbP, C3bc, sC5b-9, C5, properdin, FH, and FI in DDD versus C3GN patients. Interestingly, these two markers are also the only two markers that were found significantly different between the C3G subgroups in a previous extensive biomarker study performed by Zhang et al. [9]. The combination of lowered properdin with elevated sC5b-9 may indicate that properdin is (partly) “consumed,” i.e., used by AP convertases to facilitate formation of C5 convertases and sC5b-9; a hypothesis that is supported by reports showing more pronounced activation of the terminal complement pathway in patients with C3GN [9, 37].

Despite the variety of treatment regimens, the outcome remains relatively benign in this pediatric cohort. Only one patient progressed to kidney failure requiring kidney replacement therapy. In total, six patients (*n* = 5 DDD, *n* = 1 C3GN) had an eGFR below 90 ml/min/1.73 m^2^ at last follow-up. Of note, lower eGFR at presentation, as we observed mainly in DDD patients, has been described as a risk factor for kidney sequelae during follow-up [17]. These findings, statistically not significant due to the small group of patients, suggest a more severe presentation and outcome in patients with DDD compared to C3GN patients, and this is in line with results reported in other cohort studies [4, 17]. The overall benign outcome we described is in contrast to numerous reports in the literature pointing to a much worse outcome for C3G, with up to 50% of patients developing kidney failure within 10 years after diagnosis [4, 7, 10, 17, 37, 41]. Nevertheless, when looking at the pediatric population specifically, several reports also indicate that the outcome of children may be better than adults and that an older age of onset is associated with a higher chance of kidney failure [10, 17, 21, 25, 26, 37, 38]. An early detection and intervention in C3G and the lower presence of other, often age-related, comorbidities might contribute to this good outcome in children.

Even though the general outcome for these pediatric patients is relatively benign, proteinuria is still present in almost half of our patients despite immunosuppressive therapy (48%) and renin–angiotensin–aldosterone system inhibition (80%). This might contribute to chronic kidney disease later in life. The ongoing proteinuria as well as our findings of (persistent) complement abnormalities in time emphasize the need for targeted complement-directed therapy. The effect of the C5 inhibitor eculizumab in patients with C3G has been studied with variable results [13, 21, 42–45]. Some studies report a potential therapeutic effect of eculizumab, especially in patients with elevated sC5b-9 levels, yet no clear benefit is observed [14]. In our study, five patients were treated with eculizumab during follow-up and these patients were described separately by Oosterveld et al. [27]. Yet, no significant difference could be observed in outcome (e.g., eGFR, C3 levels, proteinuria, hypertension) when compared to the other patients who did not receive eculizumab. In contrast to atypical hemolytic syndrome, in C3G patients, it might be necessary to intervene at an earlier level in the complement cascade, for example at the level of C3 conversion. Moreover, with the marked differences in complement biomarker profiles in patients with C3GN or DDD, different complement inhibitors should be considered for subtypes of C3G patients. At this moment, a wide variety of complement therapeutics are in the pipeline and subsequently studied in various phases of clinical trials [11, 12]. Furthermore, as C3NeFs are present in the vast majority of patients, one could argue to target these autoantibodies. However, reports in the literature targeting the production of C3NeFs with anti-CD19/CD20 monoclonal antibodies such as rituximab are scarce, and the described outcome varies highly [46–48].

This study contains several limitations, mainly due to its retrospective character. The availability of patient material was limited, especially samples at presentation. Therefore, mixed model analysis was performed for the specialized complement biomarkers to include all available samples irrespective of disease state. The complement data were too limited to analyze relationships between complement marker profiles and clinical parameters. Furthermore, since there is no universal treatment scheme for patients with C3G, a highly diverse treatment regimen was represented in our study, with the choice of therapy depending on the preference of the treating physician at that time. Therefore, the effect of treatment on outcome was difficult to predict in this retrospective multicenter study.

In conclusion, this study is one of the largest C3G cohorts including solely pediatric patients and describes a follow-up period in which longitudinal complement analysis, including complement marker analysis and C3NeF detection, could be performed. Although the outcome of patients was relatively benign, persistent signs of complement (AP) dysregulation and persistent kidney injury underline the need for adequate targeted treatment to prevent decline in kidney function. Future prospective longitudinal studies are needed to further elucidate the different pathogenic mechanisms underlying this rare kidney disease, and to guide (and monitor) personalized treatment and predict outcome.

## Supplementary Information

Below is the link to the electronic supplementary material.

Supplementary file1 (DOCX 128 KB)

## Data Availability

The datasets generated and/or analyzed during the current study are not publicly available since at the time of acquiring informed consent, patients did not give permission to publish specific data sets. Additional information is available from the corresponding author on reasonable request.

## Declarations

The results presented in this paper have not been published previously in whole or part, except for abstract formats.
