# Supplementary material for: Long-term follow-up including extensive complement analysis of a pediatric C3 glomerulopathy cohort
Source: Pediatr Nephrol. 2021 Sep 2;37(3):601–12. doi: 10.1007/s00467-021-05221-6 (PMC8921070; doi:10.1007/s00467-021-05221-6)
Supplement: Supplementary file 1 — Supplementary file1 (DOCX 128 KB) [file 467_2021_5221_MOESM1_ESM.docx]

**Supplementary Information**

Belonging to:

*Pediatric Nephrology*

**Long-term follow-up including extensive complement analysis of a pediatric C3 glomerulopathy cohort**

M.A.H.M. Michels^1^ (0000-0002-0750-4493), K.L. Wijnsma^1^ (0000-0003-0019-109X), R.A.J. Kurvers^2^, D. Westra^1^ (0000-0002-5567-1704), M.F. Schreuder^1^ (0000-0001-9725-4856), J.A.E van Wijk^3^, A.H.M. Bouts^3^, V. Gracchi^4^ (0000-0002-0330-4283), F.A.P.T. Engels^2^, M.G. Keijzer-Veen^6^, E.M. Dorresteijn^7^, E.B. Volokhina^1,8^ (0000-0002-4294-8460), L.P.W.J. van den Heuvel^1,8,9^ (0000-0003-3917-6727), N.C.A.J. van de Kar^1^ (0000-0002-1990-1189)

Affiliations:

*^1^Department of Pediatric Nephrology, Amalia Children’s Hospital, Radboud university medical center, Radboud Institute for Molecular Life Sciences, Nijmegen, The Netherlands;*

*^2^Department of Pediatric Nephrology, Academic Medical Center Maastricht, Maastricht, The Netherlands;*

*^3^Department of Pediatric Nephrology, Emma Children’s Hospital, Amsterdam University Medical Center, Amsterdam, The Netherlands;*

*^4^Department of Pediatric Nephrology, Beatrix Children’s Hospital, University Medical Center Groningen, University of Groningen, Groningen, The Netherlands;*

*^6^Department of Pediatric Nephrology, Wilhelmina Children’s Hospital, University Medical Center Utrecht, Utrecht, The Netherlands;*

*^7^Department of Pediatric Nephrology, Sophia Children’s Hospital, Erasmus Medical Center, Rotterdam, The Netherlands;*

*^8^Department of Laboratory Medicine, Radboud university medical center, Nijmegen, The Netherlands;*

*^9^Department of Pediatrics/Pediatric Nephrology and Department of Development and Regeneration, University Hospitals Leuven, Leuven, Belgium.*

Corresponding author: Marloes Michels, [marloes.michels@radboudumc.nl](mailto:marloes.michels@radboudumc.nl)

**Supplementary Table 1 Genetic variants identified in the C3G cohort**

| **Patient** | **Diagnosis** | **Gene** | **DNA change (heterozygous)** | **Protein change** | **Minor allele frequency^a^** | **Literature** | **Classification^b^** | **Acquired abnormalities** |
| --- | --- | --- | --- | --- | --- | --- | --- | --- |
| P8 | DDD | *CFHR2*  (NM_005666.2) | c.595G>T | p.Glu199* | 0.75% | Not previously associated with C3G | Uncertain significance | C3NeF |
|  |  | *CFI*  (NM_000204.3) | c.1534+5G>T | p.? | 0.87% | Previously reported in aHUS [1-4] | Likely benign |  |
| P13^c^ | DDD | *CFHR5*  (NM_030787.3) | c.486dup | p.Glu163Argfs*35 | 0.20% | Previously described in C3G patients and leads to a truncated protein [5,6] | Uncertain significance | - |
| P21 | C3GN | *CFHR5*  (NM_030787.3) | c.542G>C | p.Arg181Thr | <0.1% | Not previously associated with C3G | Uncertain significance | C3NeF |

C3NeF; C3 nephritic factor, C3GN; C3 glomerulonephritis, DDD; dense deposit disease

^a^According to the gnomAD database
^b^According to the KDIGO guidelines [7]
^c^This patient also carried the *CFHR5* variant c.622T>C (p.Cys208Arg), in *cis*, but due to the frameshift earlier in the gene, this variant will likely not become functionally relevant.

**References:**

1. Caprioli J, Noris M, Brioschi S, Pianetti G, Castelletti F, Bettinaglio P, Mele C, Bresin E, Cassis L, Gamba S, Porrati F, Bucchioni S, Monteferrante G, Fang CJ, Liszewski MK, Kavanagh D, Atkinson JP, Remuzzi G, International Registry of R, Familial HT (2006) Genetics of HUS: the impact of MCP, CFH, and IF mutations on clinical presentation, response to treatment, and outcome. Blood 108 (4):1267-1279. doi:10.1182/blood-2005-10-007252

2. Sellier-Leclerc AL, Fremeaux-Bacchi V, Dragon-Durey MA, Macher MA, Niaudet P, Guest G, Boudailliez B, Bouissou F, Deschenes G, Gie S, Tsimaratos M, Fischbach M, Morin D, Nivet H, Alberti C, Loirat C, French Society of Pediatric N (2007) Differential impact of complement mutations on clinical characteristics in atypical hemolytic uremic syndrome. Journal of the American Society of Nephrology 18 (8):2392-2400. doi:10.1681/ASN.2006080811

3. Geerdink LM, Westra D, van Wijk JA, Dorresteijn EM, Lilien MR, Davin JC, Komhoff M, Van Hoeck K, van der Vlugt A, van den Heuvel LP, van de Kar NC (2012) Atypical hemolytic uremic syndrome in children: complement mutations and clinical characteristics. Pediatr Nephrol 27 (8):1283-1291. doi:10.1007/s00467-012-2131-y

4. Maga TK, Nishimura CJ, Weaver AE, Frees KL, Smith RJ (2010) Mutations in alternative pathway complement proteins in American patients with atypical hemolytic uremic syndrome. Hum Mutat 31 (6):E1445-1460. doi:10.1002/humu.21256

5. Vernon KA, Goicoechea de Jorge E, Hall AE, Fremeaux-Bacchi V, Aitman TJ, Cook HT, Hangartner R, Koziell A, Pickering MC (2012) Acute presentation and persistent glomerulonephritis following streptococcal infection in a patient with heterozygous complement factor H-related protein 5 deficiency. Am J Kidney Dis 60 (1):121-125. doi:10.1053/j.ajkd.2012.02.329

6. Figueres ML, Fremeaux-Bacchi V, Rabant M, Galmiche L, Marinozzi MC, Grunfeld JP, Noel LH, Servais A (2014) Heterogeneous histologic and clinical evolution in 3 cases of dense deposit disease with long-term follow-up. Hum Pathol 45 (11):2326-2333. doi:10.1016/j.humpath.2014.07.021

7. Goodship TH, Cook HT, Fakhouri F, Fervenza FC, Frémeaux-Bacchi V, Kavanagh D, Nester CM, Noris M, Pickering MC, Rodríguez de Córdoba S, Roumenina LT, Sethi S, Smith RJ (2017) Atypical hemolytic uremic syndrome and C3 glomerulopathy: conclusions from a "Kidney Disease: Improving Global Outcomes" (KDIGO) Controversies Conference. Kidney Int 91 (3):539-551. doi:10.1016/j.kint.2016.10.005

**
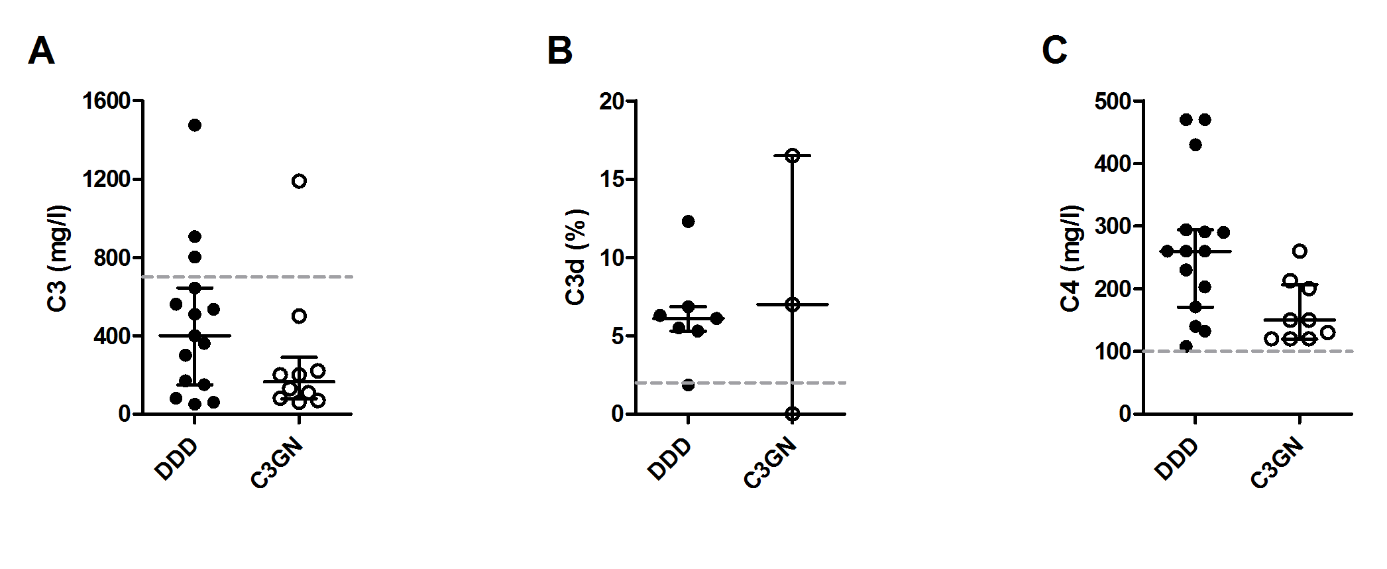
Supplementary Fig. 1 Complement markers in the acute phase**Complement C3 (A), C3d (B) and C4 (C) levels were measured in serum at diagnosis. Median and interquartile range are given. Closed dots represent samples from dense deposit disease (DDD) patients, while the open dots represent samples from C3 glomerulonephritis (C3GN) patients. Gray dotted lines indicate lower (A and C) and upper (B) cut-off values based on the mean ± 2 standard deviations of healthy controls.
